# Integrated Pedal System for Data Driven Rehabilitation

**DOI:** 10.3390/s21238115

**Published:** 2021-12-04

**Authors:** Alessandro Schaer, Oskar Helander, Francesco Buffa, Alexis Müller, Kevin Schneider, Henrik Maurenbrecher, Barna Becsek, George Chatzipirpiridis, Olgac Ergeneman, Salvador Pané, Bradley J. Nelson, Nina Schaffert

**Affiliations:** 1 Magnes AG, Selnaustrasse 5, 8001 Zurich, Switzerland; francesco.buffa@magnes.ch (F.B.); barna@magnes.ch (B.B.); chgeorge@magnes.ch (G.C.); oergeneman@magnes.ch (O.E.); 2 Institute of Robotics and Intelligent Systems, ETH Zurich, Tannenstrasse 3, 8092 Zurich, Switzerland; ohelander@student.ethz.ch (O.H.); alexis.mueller70@gmail.com (A.M.); skevin@ethz.ch (K.S.); hmaurenbrecher@gmail.com (H.M.); bnelson@ethz.ch (B.J.N.); 3 Institute für Bewegungswissenschaft, University Hamburg, Turmweg 2, 20148 Hamburg, Germany; nina.schaffert@uni-hamburg.de; 4 BeSB Sound Engineering Berlin, Nalepastraße 18, 12459 Berlin, Germany

**Keywords:** integrated pedal system, interactive training, ergometer rehabilitation, gamified rehabilitation

## Abstract

We present a system capable of providing visual feedback for ergometer training, allowing detailed analysis and gamification. The presented solution can easily upgrade any existing ergometer device. The system consists of a set of pedals with embedded sensors, readout electronics and wireless communication modules and a tablet device for interaction with the users, which can be mounted on any ergometer, transforming it into a full analytical assessment tool with interactive training capabilities. The methods to capture the forces and moments applied to the pedal, as well as the pedal’s angular position, were validated using reference sensors and high-speed video capture systems. The mean-absolute error (MAE) for load is found to be 18.82 N, 25.35 N, 0.153 Nm for $F_{x}$, $F_{z}$ and $M_{x}$ respectively and the MAE for the pedal angle is 13.2°. A fully gamified experience of ergometer training has been demonstrated with the presented system to enhance the rehabilitation experience with audio visual feedback, based on measured cycling parameters.

## 1. Introduction

A growing global population combined with higher life expectancy has increased the number of elderly people in the world to unprecedented levels. As a consequence, the demand of healthcare services and expenditures in national health services has seen a dramatic increase. Physiotherapy is a major part of these services needed by the elderly population for physical rehabilitation, injury prevention, and well-being. In Europe, the market for physiotherapy services was forecast to grow 7.7% annually from 2018 to 2023, with the global market expected to reach over $165 billion by 2023 [1]. This also increases the load on hospitals and clinics for such services. There is an immediate need for new technologies to efficiently handle the needs of the aging population.

The aim of rehabilitation is to enable a person to regain their health after an injury, disease, or surgery [2]. Successful rehabilitation leads to higher independence for the individual, decreasing the load imposed on caretakers (e.g., nursing homes) and on their families. Studies have shown that rehabilitation is most effective when it is tailored to the individual. Thus, it is of paramount importance that training programs and intervention strategies are planned on a patient by patient basis. Currently, this often is not the case, due to the high costs and limited availability of specialized caregivers [3,4].

Ergometer training has been regularly utilized for rehabilitation and is shown to provide many benefits to patients, such as increased muscle strength, reduced risk of cardiovascular disorders, and significant improvements in metabolic responses [5]. Patients suffering from neurological or physiological conditions that result in an impairment of coordination, strength or conditioning, as well as patients suffering from cardiopulmonary diseases, benefit significantly from rehabilitation with ergometer training [6,7,8]. Today, most ergometer devices do not provide the ability to provide advanced analytics, and training sessions are dull for the patient where progress is either not monitored or observed only by the total power output and the average cadence. Most rehabilitation exercises including ergometer training are repetitive and require a long-term commitment to see any benefits. However, less than half of the patients actually perform the training exercises prescribed by their therapists [9]. Psychological encouragement is important to motivate participants to train regularly, and an individual’s motivation has been shown to be strongly linked to training participation, likelihood to continue the rehabilitation, and overall performance [10,11]. Gamifying rehabilitation and athletic exercises with interactive games have been attempted to motivate patients to perform the necessary training. Video games have been utilized with success for this purpose in both athletic and rehabilitation purposes [12]. Overall, ergometer training for rehabilitation would benefit from a refined individualized training approach with a motivational stimulus.

Ergometer training is performed by a large number of athletes seeking to maximize their performance. Their post-session analyses are often more detailed, including but not limited to, tracking the power output of each leg individually, the applied forces, and the joint angles for each phase of a pedaling cycle. This detailed analysis allows athletes to fine tune their training sessions to specifically work on weaknesses and imbalances for better performance. The recent tools and technologies utilized by athletes have not been adapted by rehabilitation programs for patients. This is mainly due to complexity in integrating different systems, and costs associated with upgrading equipment.

In this work, we present a system capable of providing advanced feedback for ergometer training, allowing for detailed analysis and gamification. The presented solution can easily upgrade any existing ergometer device. The system consists of a set of pedals with embedded sensors, readout electronics and a wireless communication module, which can be mounted on any ergometer. It will transform the ergometer into a full analytical assessment tool with interactive training capabilities. A complete analysis of ergometer training can be performed by capturing training parameters not measured by standard ergometers. The system also allows for the gamification of the rehabilitation exercises due to the large number of captured parameters. By measuring the user’s output continuously and giving feedback to the user with an appended tablet device, the pedals can be used as controllers to play a game on the tablet. Our system augments the rehabilitation experience by giving a motivational stimulus, through the gamification of the training process and providing in-depth analytics.

**Figure 1:**
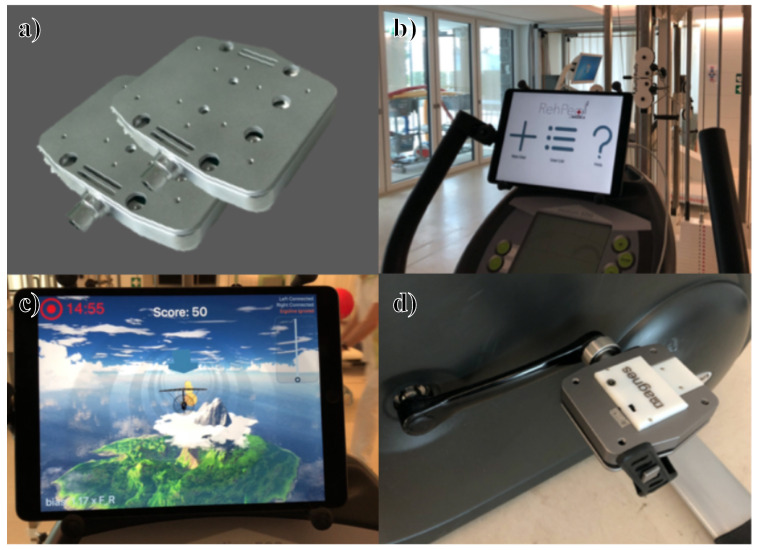
The developed ergometer-upgrade system. (**a**) 3D rendering of the sensor-equipped pedals; (**b**) The tablet mounted on an ergometer displaying the developed app’s home view; (**c**) In-game view of a training session; (**d**) One of the pedals installed on the ergometer—here, the pedal is upside down.

See Figure 1 for a depiction of the developed system. The contribution of the design is of practical nature, meaning that the ultimate goal is to perform experiments with the developed system and assess its contribution to the rehabilitation process.

This paper is structured as follows; we first describe the pedal system and present its operation principle. We evaluate a method to extract the applied forces and torques compared to from raw sensor data. We then explore the methods with which a patient’s cycling parameters are estimated. Specifically, the methods for estimating the pedal and crank angle are presented. The former is of importance as it is closely linked to the foot’s ankle angle, a key metric for assessing a person’s joint control. Crank angle, on the other hand, provides the ability to compare the consistency of one’s pedaling patterns during a session, allowing for a more refined post-session analysis. We then give an overview of all analytical outputs, which are generated by the system. Lastly, an implementation of an interactive training program utilizing the pedal system and a game displayed on a tablet device is presented.

## 2. Hardware

**Figure 2:**
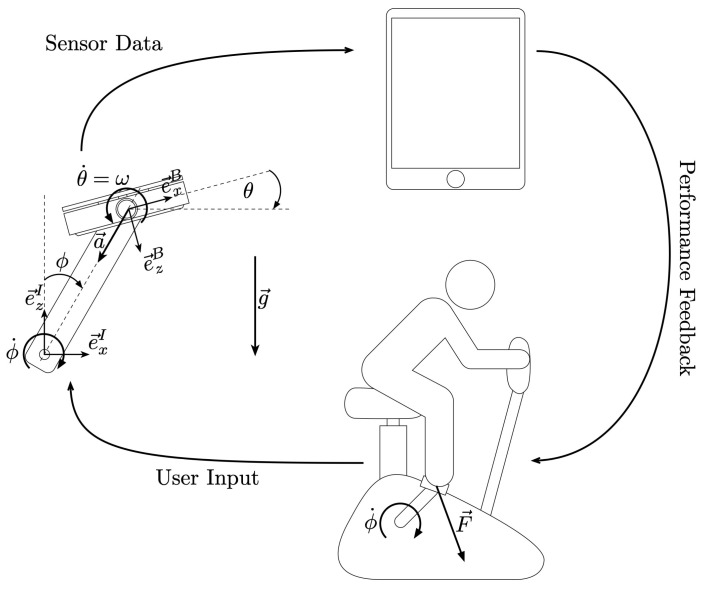
Graphical illustration of the pedal system. Users cycle on the ergometer, applying a force on the pedals. The pedals measure the load applied and transfers it to a tablet device for analysis. Audio or visual feedback is transmitted back to the user to provide feedback.

The developed pedals comprise an inductive sensor measuring the applied load and an inertial measurement unit (IMU) consisting of an accelerometer and a gyroscope. The pedal’s sensor suite thus measures the experienced load, acceleration, and angular velocity each along three axes. A full breakdown of the components in the pedal system can be seen in Figure 2. The system further includes: a nRF52832 (Nordic Semiconductor, Trondheim, Norway) SoC running a custom C firmware handling sensor readout and BLE data communication to a smartphone/tablet; a rechargeable, single cell LiPo battery; and a battery charger.

Accelerations and angular velocities are measured using a BMI160 (Bosch, Robert Bosch GmbH, Gerlingen, Germany) 6D, 16-bit IMU. The readout IC for our force sensing solution is a LDC1614 (Texas Instruments Inc., Dallas, Texas, USA) 4-channel, 28-bit, inductance-to-digital converter.

Our inductive sensor consists of a copper plate (target) and an inductive coil wired in parallel with a capacitor. This creates an LC resonant circuit with variable inductance *L*, which we refer to as LC-tank. By changing the relative position of the target and coil, the resonance frequency of the LC-tank shifts as a consequence of Faraday’s law of induction [13,14].

**Figure 3:**
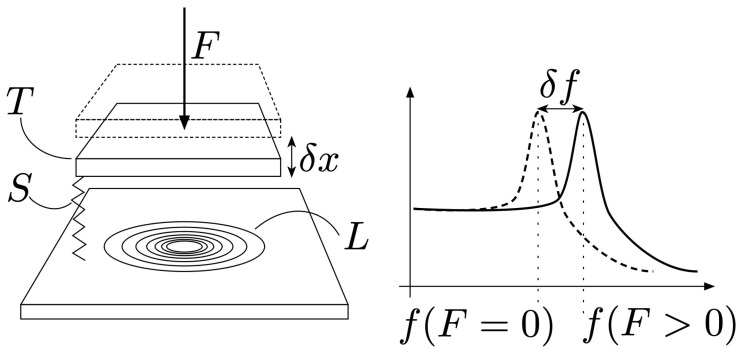
Load sensor working principle. By applying a force *F* to the target *T*, the spring *S* is gets displaced by δx. This change in target position causes the inductance *L* of the LC-tank to change, and thus causes a change in resonance frequency δf. By measuring the resonance frequency *f* of the LC-tank for various forces *F*, one can construct a mapping from *f* to *F* and thus estimate the forces based on the LC-tank’s resonance frequency.

By measuring the resonance frequency of the LC-tank, the distance between the target and the coil can be calculated. The target is mounted on a spring, so the load applied to the target can be translated into displacement, and hence, with the LC-tank into a change in resonance frequency. This process is illustrated in Figure 3. By utilizing four LC-tanks and placing them in a certain orientation with respect to the target, the displacement of the target in three axes can be calculated. Loads exerted along three axes can be measured using this method.

**Figure 4:**
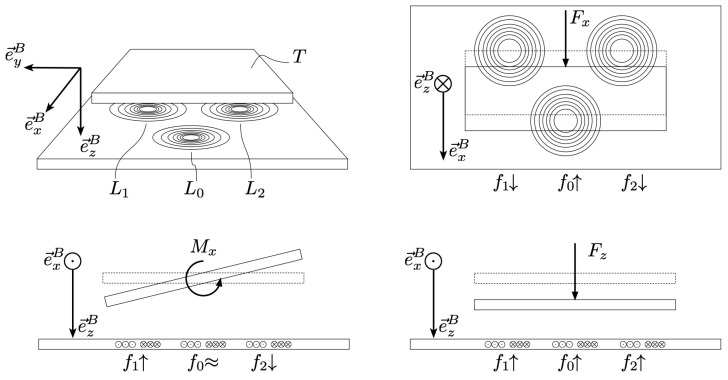
Working principle for 3D load detection using three coils. Here, a forward shear force $F_{x}$ will cause a change in $f_{0}$, and the opposite change in $f_{1}$ and $f_{2}$. Torque around the *x*-axis $M_{x}$ would cause a change in $f_{1}$ and the opposite change in $f_{2}$, with $f_{0}$ remaining constant. Finally, a normal force $F_{z}$ will cause all frequencies to change equally. With this, all load types can be differentiated.

A spring that deforms in 3D was designed to measure the most significant forces and torques for the intended application, i.e., pedalling. These are the normal force $F_{z}$, the forward shear force $F_{x}$ and torque around the *x*-axis $M_{x}$. The shear forces in *y*-direction, and the torques in *y*- and *z*-direction, are either negligible or not of interest for the intended application. The configuration of the coils and a qualitative depiction of the behavior of the resonance frequencies as a function of the three typical load cases can be seen in Figure 4.

## 3. Methods

### 3.1. Definitions and Notation

In this section, we explain the calibration procedures used for obtaining the desired information from the raw sensor data. First, we illustrate the procedure for mapping LC-tanks resonance frequencies $\vec{f}$ to load values $\vec{F}$. Second, we show how we get the kinematic parameters of interest for our application. The kinematic parameters of interest are the crank angle ϕ, the pedal angle θ, and the cadence $\dot{\phi }$. Everything reported herein applies to both hand-sides, left and right, but each side is evaluated independent from the other. For details on the used notation, please refer to the Appendix A.1.

#### 3.1.1. Crank Angle Definition

**Figure 5:**
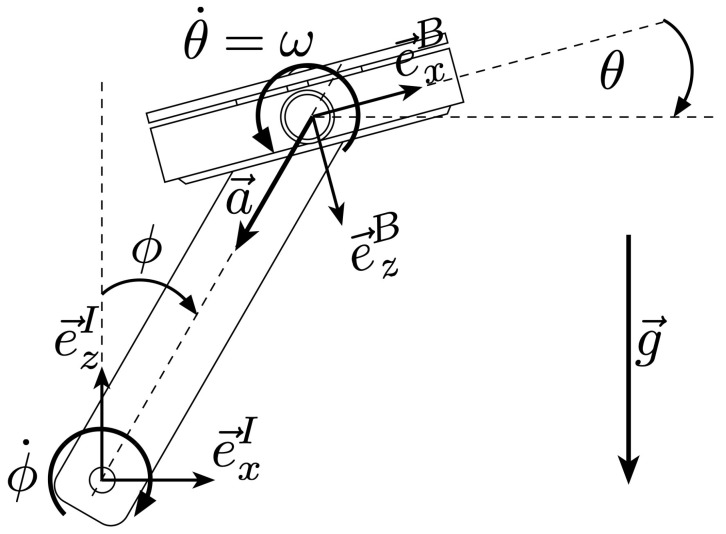
Pedal kinematics modeling. Annotated definition of the world frame *I*, body frame *B*, crank angle ϕ and pedal angle θ. Note that the pedal angle θ is defined with respect to the world-horizontal plane (perpendicualr to the gravity vector $\vec{g}$) and is independent of the crank-angle ϕ. The gyroscope measures the pedal’s angular rate $\dot{\theta }=\omega$, while the accelerometer measures the pedal’s acceleration biased by gravity ${}_{B}\ddot{\vec{x}}={}_{B}\vec{a}+{}_{B}\vec{g}$. Please also note that both frames of reference *I* and *B* are 3D orthonormal, right-handed frames, with their *y*-axis pointing inward and outward respectively. These axes are not depicted in the image to avoid overcrowding. The pedal’s motion is mechanically constrained to the xz-plane, and it can thus be assumed, without loss of generality, that the *y*-position of the pedal is constant at 0.

We define the world frame *I* to be centered on the axle of the crank arm with the *x*- and *y*-direction parallel to the floor, with the *x*-direction pointing towards the ‘direction of cycling’, and the *y*-direction pointing left. The *z*-axis is normal to the ground and pointing upwards. The crank angle ϕ is defined as the angle between the *z*-axis of the world frame, and the line connecting the crank axle with the pedal’s axle. See Figure 5 for reference.

#### 3.1.2. Pedal Angle Definition

The pedal body frame *B* is centred on the axis of rotation of the pedal with the *x*-direction running towards the ‘direction of cycling’. The *z*-axis normal to the surface of the pedal pointing downwards when the pedal’s *x*-axis is aligned with the world frame’s *x*-axis. The pedal angle θ is defined as the angle between the *x*-axis in the pedal frame and the *x*-axis of the world frame Figure 5. According to our definition, the pedal angle is expected to be constrained to a subrange of $\left[-90^{{}^{\circ}},90^{{}^{\circ}}\right]$ depending on the user’s ankle flexibility.

### 3.2. Load Sensor Calibration

We calibrate each axis of the load sensor individually by collecting data with our sensor and a reference sensor (OMD-45-FH-2000N; OptoForce Ltd., Budapest, Hungary) having 1N resolution and 1000N max (compressive) load. We map the resonance frequency readouts $\vec{f}$ to the load readouts $\vec{F}$ using linear machine learning models and cross-validation.

**Figure 6:**
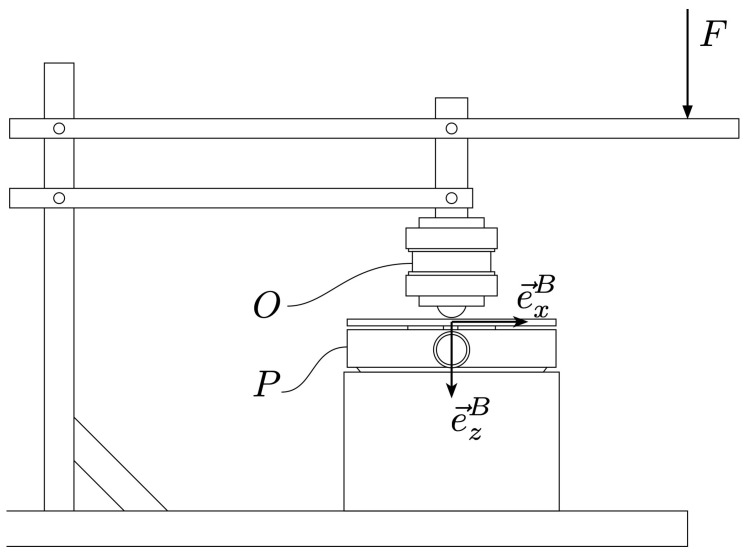
Schematic representation of the force-calibration setup. Depending on the mounting mode of the pedal *P*, the operator can apply forces *F* to *P* and collect, simultaneously, data coming from *P* and the reference sensor *O*. Three mounting modes are possible, enabling loading in $F_{x}$, $F_{z}$, and $M_{x}$.

A custom calibration setup was built for collecting calibration data. The calibration setup consists of a mounting tower, a reference sensor, and a load-lever. The calibration foresees an operator handling the data acquisition with the two sensors and applying loads to the system. In the following, we detail the calibration protocol and the models used for mapping from frequency-readouts to force/torque readouts. A schematic representation of the calibration setup is depicted in Figure 6.

For each desired output $F\in U=\{F_{x},F_{z},M_{x}\}$, the sensor is mounted on the calibration device such that the dominant applied load is the desired output. After both systems, reference sensor and pedal, have started logging, the system is loaded eight times in cycles within the calibration range. We denote with T={k:k∈calibrationtime} the set of all time samples occurring during the calibration run. We obtain two datasets $I=\{\vec{f}\left[k\right]:\vec{f}=\left(f_{0}\left[k\right],f_{1}\left[k\right],f_{2}\left[k\right],f_{3}\left[k\right]\right)\;\forall \;k\in T\}$ and F={F[k]:F[k]∈U∀k∈T} having a one-to-one correspondence mapped by

(1) $$M:I\to F,\vec{f}\left[k\right]\mapsto F\left[k\right]=M\left(\vec{f}\left[k\right]\right)+e\left[k\right],$$

where e[k] denotes the estimation error. From here on, we drop the time sample *k* dependence, as the mapping does not depend on time, since it is algebraic.

Thus, we define the three estimates ${\hat{F}}_{x}$, ${\hat{F}}_{z}$, and ${\hat{M}}_{x}$ to be

(2) $$\begin{array}{cc} {\hat{F}}_{x} & =M_{x}\left(\vec{f}\right), \end{array}$$

(3) $$\begin{array}{cc} {\hat{F}}_{z} & =M_{z}\left(\vec{f}\right), \end{array}$$

(4) $$\begin{array}{cc} {\hat{M}}_{x} & =M_{M}\left(\vec{f}\right). \end{array}$$

As mappings $M_{i}$, we use cross-validation LASSO estimators. The models are trained using scikit-learn [15] with 10-fold cross-validation and 30% test-fraction, and a 25-elements regularization log-space from $10^{-10}$ to $10^{2}$.

### 3.3. Pedalling Kinematics

#### 3.3.1. Pre-Processing

In order to limit the effect of noise and improve results, the raw data were low-pass filtered before being further processed. All analysis relevant signals have been passed through a second order Butterworth [16] low-pass filter with the cutoff frequency set at $f_{C}=2.2\;\mathrm{Hz}$. The filter was applied using the forward-backward filtering filtfilt() function implemented in the scipy.signal [17] Python module.

#### 3.3.2. Kinematic Model

For slowly changing cadences, i.e., $\ddot{\phi }\approx 0$, we can write the acceleration of the pedal represented in the world frame ${}_{I}\ddot{\vec{x}}$ as

(5) $${}_{I}\ddot{\vec{x}}=\left[\begin{array}{c} -r{\dot{\phi }}^{2}\sin\left(\phi \right)\\ -r{\dot{\phi }}^{2}\cos\left(\phi \right)-g \end{array}\right]$$

where *r* is the crank-arm length, i.e., the distance between the crank-axle-center and the pedal-axle-center, and $g=9.81\;m/s^{2}$ is the gravitational acceleration. By applying the rotation matrix $R_{BI}$ to the acceleration, we get the theoretical output of the IMU as

(6) $${}_{B}\ddot{\vec{x}}=R_{BI}\cdot {}_{I}\ddot{\vec{x}}=\left[\begin{array}{c} -r{\dot{\phi }}^{2}\left(\sin(\phi )\cos(\theta )+\cos(\phi )\sin(\theta )\right)-g\sin\left(\theta \right)\\ -r{\dot{\phi }}^{2}\left(\sin(\phi )\sin(\theta )-\cos(\phi )\cos(\theta )\right)+g\cos\left(\theta \right) \end{array}\right].$$

For more details on the derivation of these results, the interested reader is referred to Appendix A.

#### 3.3.3. Crank Angle Estimation

The squared magnitude of the acceleration ${\ddot{x}}^{2}$ for crank revolutions performed at approximately constant rates can be expressed as

(7) $$a^{2}:={\ddot{x}}^{2}={\left|\right|}_{I}\ddot{\vec{x}}{\left|\right|}_{2}^{2}={\left|\right|}_{B}\ddot{\vec{x}}{\left|\right|}_{2}^{2}=r^{2}{\dot{\phi }}^{4}+2gr{\dot{\phi }}^{2}\cos\left(\phi \right)+g^{2}$$

it can be seen that this is maximal when the crank-arm is at top dead center (TDC) $\phi =0^{{}^{\circ}}$ and minimal at bottom dead center (BDT) $\phi =180^{{}^{\circ}}$. Given that, for our application, the approximation for constant cadence $\dot{\phi }\approx 0$ is reasonable, this allows us to detect the crank angle at TDC and BDC by identification of the maxima and the minima of the acceleration magnitude signal $\ddot{a}\left(t\right)$. To obtain the crank angle ϕ(t) for every time-stamp *t*, we apply linear interpolation for all points between successive maxima and minima. This approach is aligned with the assumption of slowly changing cadences $\ddot{\phi }\approx 0$. Our assumption is thus that each individual crank revolution is carried out at a constant rate, but steady-state pedalling is not required i.e., a constant cadence throughout the measurement session is not required.

To validate this method, we check the offset between the left and right pedal: ideally, the crank angle should be offset by $\Delta \phi_{LR}=180^{{}^{\circ}}$ between the two sides. This is done by taking the crank angle of one side for a specific time stamp and computing the offset with that of the measured value on the other side. Since the time stamps are not perfectly synchronized, linear interpolation is done between the value of the crank angle of the closest time stamp before and after the value in question to estimate the value of the crank angle on the other side. The average offset for the session is then taken. The average crank angle offset is computed to be $\Delta \phi_{LR}=175.75^{{}^{\circ}}\pm 0.920^{{}^{\circ}}$.

#### 3.3.4. Pedal Angle Estimation

**Figure 7:**
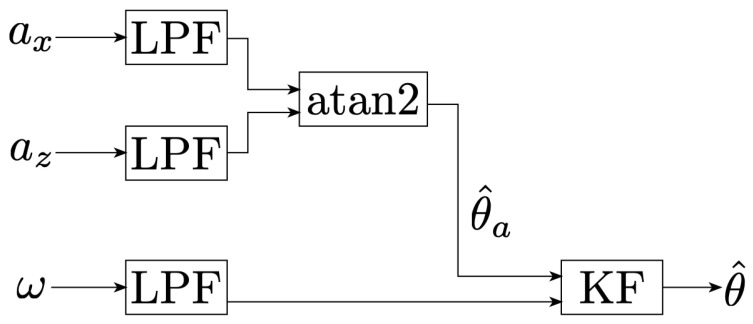
Flow chart outlining the process used to derive the pedal angle. After passing the analysis-relevant signals through second order Buttwerworth low-pass filter (LPF), we compute a rough estimate of the pedal angle based on accelerometer measurements ${\hat{\theta }}_{a}$ and then fuse the gyroscope measurements ω with ${\hat{\theta }}_{a}$ using a KF to get a refined version of the estimate $\hat{\theta }$.

The overall pedal angle estimation method is detailed in Figure 7. The method consists in a first rough estimation from the accelerometer measurements and then feeding this rough estimate to a Kalman filter (KF), in which the gyroscope is integrated. This method has already been proposed in [18] while using high sampling rates ($f_{s}=500\;\mathrm{Hz}$). Since our sensors sample at $f_{s}=25\;\mathrm{Hz}$, the method’s effectiveness at these lower frequencies has to be evaluated.

##### Rough Estimate—Acceleration Angle

We compute a first rough pedal angle estimate ${\hat{\theta }}_{a}\left(t\right)$ using the accelerometer measurements:

(8) $${\hat{\theta }}_{a}\left(t\right)=\arctan2\left({}_{B}{\ddot{x}}_{x},{}_{B}{\ddot{x}}_{z}\right).$$

This estimation is exact for the case in which $\dot{\phi }=0\;\forall \;t$, as can be seen from the following equation:

(9) $${}_{B}\ddot{\vec{x}}=\left[\begin{array}{c} -g\sin(\theta )\\ g\cos(\theta ) \end{array}\right].$$

However, this estimate is only accurate when either the crank arm is stationary $\dot{\phi }=0$ or when the crank arm is at $\phi \in \{0^{{}^{\circ}},\;90^{{}^{\circ}}\}$, as can be seen in (6). We can define the uncertainty of this ${\hat{\theta }}_{a}$ as ρ(t), and thus write

(10) $${\hat{\theta }}_{a}\left(t\right)=\theta \left(t\right)+\rho \left(t\right).$$

##### Refining the Estimation—Kalman Filter

As the pedals feature a gyroscope, we can also estimate the pedal angle by integrating the angular velocity $\omega \left(t\right)=\dot{\theta }$ signal of the pedal. Dead reckoning is a notoriously difficult task, and naively integrating noisy IMU data is as unreliable as the acceleration-based estimation previously introduced [18]. But by fusing the two approaches by means of a KF, an improved estimate for the pedal-angle $\hat{\theta }$ can be obtained.

The KF fuses the data received from the accelerations and that of the angular velocity. The KF [19] gives the best estimate of the pedal angle by accounting for noise, by approximating the sensor data output as Gaussian distributions.

As done in [18], we define the underlying state space model used in our KF implementation as follows:

(11) $$\begin{array}{cc} \hat{\theta }\left[k\right] & =\hat{\theta }\left[k\right]+\omega \left[k\right]\cdot T_{s}+\nu \left[k\right]\\ y[k] & =\theta \left[k\right]+\rho \left[k\right]={\hat{\theta }}_{a}\left[k\right] \end{array}$$

where we use the discrete sample time [k], $T_{s}$ is the sampling interval, ν(·)∼N(0,Q) is the process noise and ρ(·)∼N(0,R) is the measurement noise (comprehending both actual noise and rough estimate uncertainty due to pedalling). It shall be noted that the assumption of ρ(·)∼N(0,R) is strong and does not reflect the reality of the system, as the uncertainty due to pedalling is actually correlated. Nevertheless, this is the simplest model, and the goal herein is to investigate what the limits of this simplification are.

## 4. Calibration Results

For any estimate $\hat{x}$ of a reference value *x*, we define the estimation error to be $e_{x}=x-\hat{x}$. We further denote the average error of said estimate with $\mu_{x}=E\left[e_{x}\right]$, and the error standard deviation with $\sigma_{x}$. The mean absolute error (MAE) is defined to be ${\mathrm{MAE}}_{x}=E\left[|e_{x}|\right]$ and the root mean squared error (RMSE) is ${\mathrm{RMSE}}_{x}=\sqrt{E\left[e_{x}^{2}\right]}$.

### 4.1. Load

**Figure 8:**
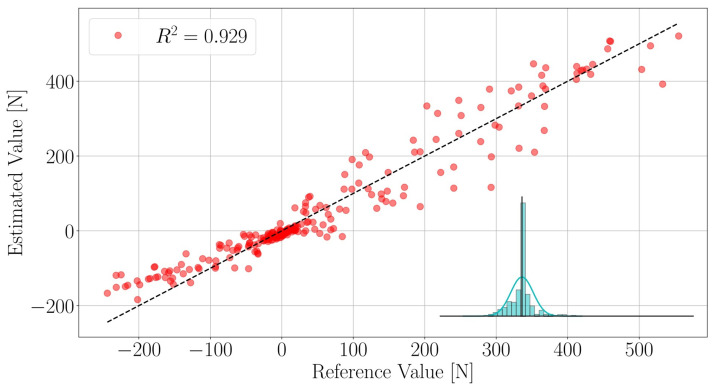
Force $F_{z}$ model performance on test data set. The histogram represents the error $e=F_{z}-{\hat{F}}_{z}$ distribution—the black vertical line is located at e=0, the bins have size 6.15N.

We execute the force calibration procedure for all loads of interest and obtain three linear models relating the resonance frequencies $\vec{f}$ of the LC-tanks to the loads $\vec{F}$. That is one model for each load. The performance of the $F_{z}$ model on the test-data-set is shown in Figure 8. The full force calibration results statistics are reported in Table 1. The proposed models are capable of reliably mapping the resonance frequencies $\vec{f}$ to the loads of interest.

### 4.2. Kinematic Parameters

**Figure 9:**
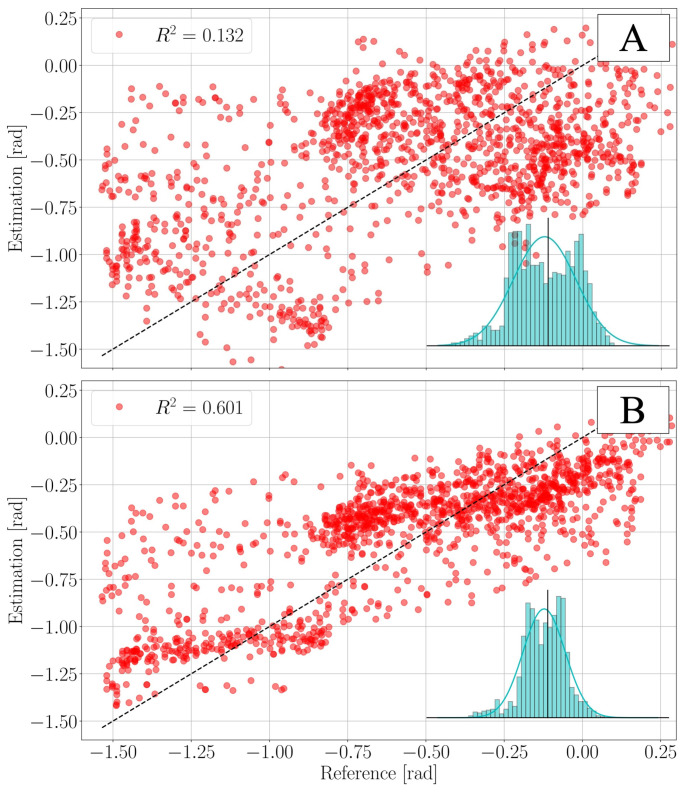
Visualization of the improvements brought by the KF for the pedal angle estimate $\hat{\theta }$. The plots above show how the pedal angle estimate $\hat{\theta }$ compares to the reference values θ extracted from the video. The histograms show the error $e=\theta -\hat{\theta }$ distributions, the vertical line is located at $e=0^{{}^{\circ}}$, and the bell curves are the Gaussian distributions N(μ(e),σ(e)), with μ(e) and σ(e) being the average error and the error standard deviation respectively. $R^{2}$ is the coefficient of determination. (**A**) accelerometer-only estimation ${\hat{\theta }}_{a}$; ${\mathrm{RMSE}}_{\theta_{a}}=24.65^{{}^{\circ}}$; ${\mathrm{MAE}}_{\theta_{a}}=21.07^{{}^{\circ}}$; $\mu_{\theta_{a}}=-2.74^{{}^{\circ}}$; $\sigma_{\theta_{a}}=24.50^{{}^{\circ}}$. (**B**) KF-estimation $\hat{\theta }$; ${\mathrm{RMSE}}_{\theta }=16.72^{{}^{\circ}}$; ${\mathrm{MAE}}_{\theta }=13.20^{{}^{\circ}}$; $\mu_{\theta }=-2.85^{{}^{\circ}}$; $\sigma_{\theta }=16.48^{{}^{\circ}}$.

The final pedal angle estimation $\hat{\theta }$ for the validation dataset has the following error $e=\theta -\hat{\theta }$ statistics: root mean squared error ${\mathrm{RMSE}}_{\theta }=16.72^{{}^{\circ}}$; mean absolute error ${\mathrm{MAE}}_{\theta }=13.20^{{}^{\circ}}$; average error $\mu_{\theta }=-2.85^{{}^{\circ}}$; error standard deviation $\sigma_{\theta }=16.48^{{}^{\circ}}$; and coefficient of determination $R_{\theta }^{2}=0.601$. It shall be noted that the performance is suboptimal if compared to the one presented in [18]. This is mainly due to two facts. First, the sampling rate used by the IMU is only 25 Hz. The system would greatly benefit from higher sampling rates, but this is problematic from a raw-data transmission point of view, as the bandwidth of the used BLE protocol is limited. Second, another source of error is to be identified in the assumption of ρ being normally distributed ρ(·)∼N(0,R). While this might well be a good approximation for the sensor-noise, it certainly does not reflect the character of human-pedalling. As a consequence, the residual estimation error is not normally distributed, as can be seen in Figure 9. Despite these short-comings, it is worth noticing that the KF improves the estimate of the pedal-angle $\hat{\theta }$ significantly compared to the accelerometer-based estimate ${\hat{\theta }}_{a}$. The validation dataset was collected using video footage at 120 fps to track the orientation of the pedal angle throughout a session. With this, the parameters of the KF were adjusted to increase accuracy. A comparison between the acceleration-based estimate ${\hat{\theta }}_{a}$ performance and the KF estimate $\hat{\theta }$ is shown in Figure 9. It should be noted that the majority of the error is attributed to areas near the extrema of the extracted pedal angle. This corresponds to moments where large changes in pedal angle are seen decreasing accuracy at low frequencies and regions where the rough pedal angle estimate ${\hat{\theta }}_{a}\left(t\right)$ is least accurate, i.e., $\phi \in \left\{90^{{}^{\circ}},270^{{}^{\circ}}\right\}$. Tracking of the peaks can be improved with a dynamic noise covariance and higher sampling rates.

## 5. Applications

We present a system that allows for a refined analysis of ergometer training exercises. This system is interchangeable with any ergometer device, allowing for it to be seamlessly integrated to any ergometer training set up for individual or clinical use. The presented system can extract individuals cycling parameters in real time, and it allows for a comprehensive data visualization and gamification of ergometer exercises. Possible implementation of data visualization and gamification is given in the next two subsections.

### 5.1. Data Visualization

In order to provide a database and data visualization tool for individuals and doctors, an iPad (Apple Inc., Cupertino, CA, USA) app was developed. This allows for a live feed of a patient’s current pedalling performance (forces and cadence), as well as a gamified experience to individuals while conducting a session. Additionally, the data collected during the sessions are stored for future use by the therapist; in particular, means for visualizing the performance of the patient are implemented.

**Figure 10:**
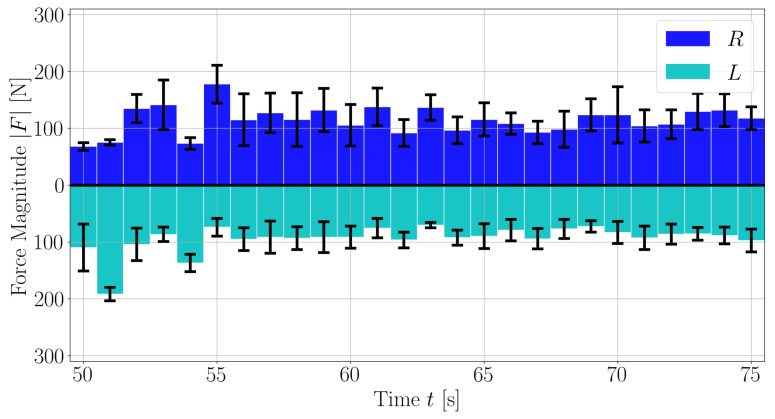
Average magnitude of the force exerted on pedal over time. Here, a section of a large assessment is shown. The averages are computed over 1s bins. The bars’ heights are the average force magnitude over the bin, and the error bars span plus and minus one standard deviation.

**Figure 11:**
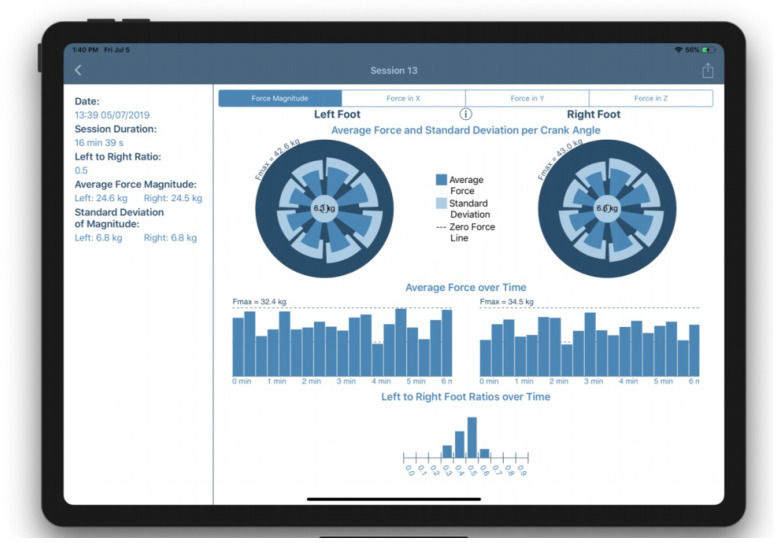
Pedalling forces breakdown. Post-session analysis showing breakdown of forces for different crank angles.

**Figure 12:**
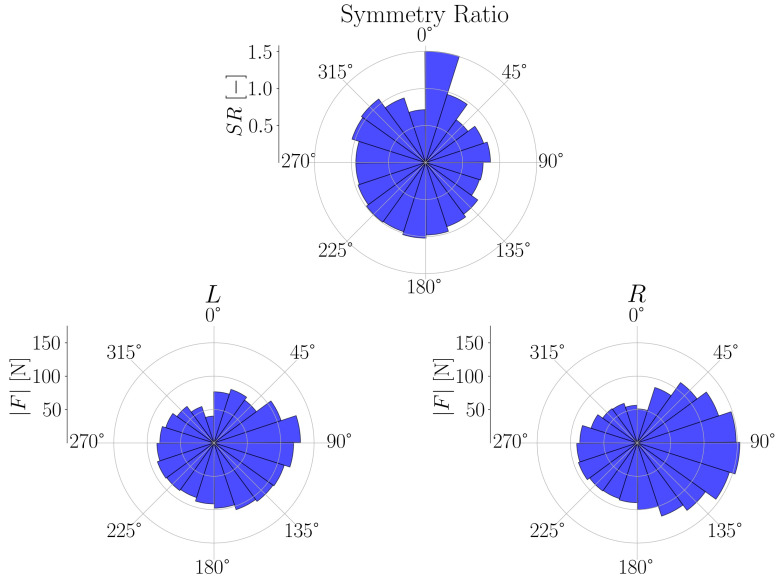
Pedalling force and force symmetry over crank angle. Histogram of the magnitude of force exerted on pedal as a function of the crank angle averaged out throughout the session.

The metrics include commonly used features such as cadence, as well as lesser used metrics such as the normal force, shear force or pedal angle to be shown for the session as a whole or for different regions of the session for left and right foot separately Figure 10. With the crank angle, we can also provide insights of these parameters for different portions of the pedalling phase, like illustrated for the force magnitude in Figure 11 and Figure 12. The bio-mechanics of cycling for athletes is well known and such information could prove to be useful when diagnosing and treating patients. This is largely due to how the general profile of these parameters as a function of the crank angle is linked to features such as the work done by certain muscle groups, joint torques, symmetry between the left and right side, and overall performance [20,21].

### 5.2. Gamification

Using the presented pedal system and a tablet device for providing audio-visual feedback, a gamified experience was realized to add a motivational aspect for physical rehabilitation. This system can be utilized for providing a personalized training experience by actively tracking certain cycling parameters and adjusting the training settings to specifically improve those parameters.

In the developed app, while the patient is pedalling, their motions control a kite flying along a trajectory. The cadence controls the speed of the kite and the force ratio between the left, and the right foot controls the yaw of the kite (e.g., if the total force exerted on the left pedal is higher than that of the right, the kite will yaw towards the left).

The patient encounters a path to follow on the screen, and by controlling their cycling parameters, they try to follow this path. A number of circuits have been designed which the patients can play in. In addition to following the circuit, the patients have to collect golden coins along the way. The more coins they collect, the higher their final score. The coins are generated along the path and stimulate the users to take more control over the kite’s position, improving the balance. The circuits are essentially 2D paths rendered in a 3D world. In order to avoid the users to go adrift, the kite’s position is constrained by an (invisible) tube along the path.

Depending on the type of therapy, one could modify the controls of the kite in the app so that different features (force, cadence, pedal angle, etc.) take control over specific game parameters. For example, a patient with significant foot-drop could be motivated by coupling their pedal angle with the kite’s pitch angle, and the positioning of coins in the top part of the circuit-tube.

**Figure 13:**
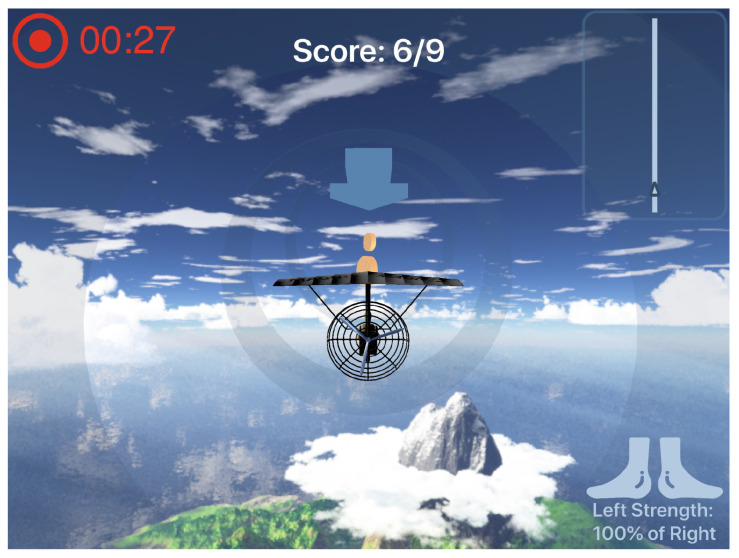
Game live-view. Render of the live-view of the game with the kite in the middle following a straight track.

**Figure 14:**
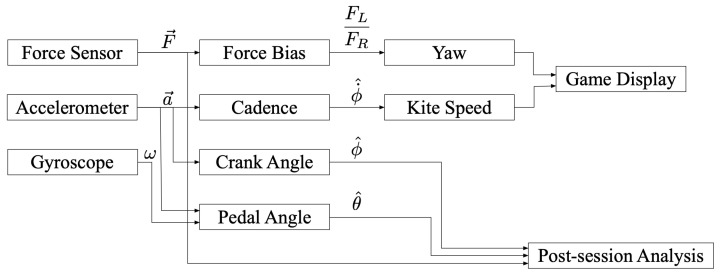
Game back-end logic depiction. This flowchart illustrates the signals flow running on the tablet while the patient is ‘playing’.

A rendering of the live-view of the game can be found in Figure 13. An overview of the methods used for the post session analysis as well as the gamified display is seen in Figure 14.

## 6. Results and Discussion

### 6.1. Conclusions

We presented an advanced ergometer rehabilitation system, which provided a detailed analysis of exercises, and enables more interactive training sessions with audio-visual feedback. We studied the sensing characteristics of the system. The methods for extracting the forces and torques applied to the pedal along with the angular position of the pedal have been evaluated using their respective reference datasets. The MAE load estimation errors are found to be 18.82 N, 25.35 N, 0.153 Nm for $F_{x}$, $F_{z}$ and $M_{x}$ respectively. The use of the linear KF decreased both the RMSE of the estimated angle and the standard deviation of the error of the estimated angle by 32.2% and 32.8% respectively from that of the rough estimate. We successfully demonstrated the usage of the developed system on an ergometer, and explored the limits of the used models. This first implementation will serve as a benchmark for future improvements. Force and cadence measurements can be used for providing feedback to the user in the game, while the pedal angle can be used for qualitative feedback, due to its relatively higher error.

We also presented a new data visualization interface, giving more insight into ergometer training sessions. An app was developed, which utilizes the feedback from the sensing system in order to gamify rehabilitation exercises. With the system in place, the effectiveness and response of patients to the gamified experience need to be further explored. This includes, but is not limited to, the importance of the visual and audio component of the game, and its ability to steer patients to cycle optimally in individual sessions and over multiple sessions in time.

### 6.2. Outlook

The area where the system exhibits the largest room for improvement is the pedal angle estimation. The used KF improves the naive estimate obtained from the accelerometer-only ${\hat{\theta }}_{a}$ significantly, but still sub-optimally. We believe that the main reasons for this are the low sampling rate of 25 Hz and the strong assumption of ρ∼N(0,R). More comprehensive estimators, such as an extended Kalman filter or an unscented Kalman filter (UKF) [22], shall be implemented to achieve better results. In particular, the UKF is promising, as it allows for a sampling of the actual uncertainty, rather than having to assume Gaussian random variables.

It will be interesting to investigate whether our simplification of constant-rate crank revolutions is robust enough for rehabilitation applications. While this is a reasonable assumption for healthy users pedalling regularly, it might not be a good approximation for impaired users who might display nonlinear crank-revolution patterns, which are potentially not approximated well enough by our linear model.

Including the ergometer’s dynamics into the model in order for it to better map the nonlinearities due to the pedalling of the user and the response of the ergometer is another path for improvement for the presented system. Ergometers possess safety features such as viscous-feedback forces, and the coupling of these dynamic effects with the user could be an interesting research topic. The modeling of these effects could improve the presented system’s accuracy.

We developed the system with real-time, gamified feedback to have an impact on rehabilitation. Due to its design it can upgrade any existing ergometer. Rehabilitating patients are going to benefit from this device. The future goal is to quantify the benefits brought by the system to actual patients. This shall be achieved by performing clinical trials where patients’ performance is monitored over extended periods.

## Figures and Tables

**Table 1:** Force sensor calibration results.

Estimate $\hat{\xi }$	Load		
	$F_{x}$ [N]	$F_{z}$ [N]	$M_{x}$ [Nm]
$\mu_{\xi }$	$2.596\times 10^{-11}$	$5.072\times 10^{-12}$	$1.576\times 10^{-12}$
$\sigma_{\xi }$	35.51	38.84	0.260
${\mathrm{MAE}}_{\xi }$	18.82	25.31	0.153
${\mathrm{RMSE}}_{\xi }$	35.51	38.84	0.260
$R_{\xi }^{2}$	0.772	0.929	0.937
$\alpha_{\xi }$	$3.16\times 10^{-5}$	$1.00\times 10^{-5}$	$3.16\times 10^{-8}$

Notes: *μ* is the average error; *σ* is the error standard deviation; MAE is the mean absolute error; and RMSE is the root mean square error. The calibration ranges (ground-truth data) are *F*_*x*_ ∈ [−189.72, 243.81] N, *F*_*z*_ ∈ [−244.12, 555.28] N, and *M*_*x*_ ∈ [−2.618, 3.329] Nm. *R*^2^ and *α* are the coefficient of determination and the LASSO regularization parameter respectively, and are both unit-less.

## Data Availability

Not applicable.

## Abbreviations

The following abbreviations are used in this manuscript:

BDC	Bottom dead center
fps	Frames per second
IC	Integrated circuit
IMU	Intertial measurement unit
KF	Kalman filter
LPF	Low-pass filter
MAE	Mean absolute error
RMSE	Root mean squared error
SoC	System on chip
TDC	Top dead center

## Appendix A. Derivation of Pedal Kinematics

### Appendix A.1. Notation

We denote $\hat{x}$ as the estimate of *x*, and $\dot{x}$ denotes the time-derivative of *x*. Let $\vec{r}\in R^{3}$ be a 3D column vector. Then, ${}_{A}\vec{r}=\left(x,y,z\right)=x\cdot {}_{A}{\vec{e}}_{x}^{A}+y\cdot {}_{A}{\vec{e}}_{y}^{A}+z\cdot {}_{A}{\vec{e}}_{z}^{A}$ with x,y,z∈R is the representation of $\vec{r}$ with respect to the orthonormal frame of reference *A* with the basis vectors ${}_{A}{\vec{e}}_{x}^{A}=\left(1,0,0\right)$, ${}_{A}{\vec{e}}_{y}^{A}=\left(0,1,0\right)$ and ${}_{A}{\vec{e}}_{z}^{A}=\left(0,0,1\right)$, and $r=\left|\right|\vec{r}\left|\right|=\sqrt{{\vec{r}}^{T}\vec{r}}$ is its 2-norm, which is invariant w.r.t. the chosen orthonormal frame of reference.

Let $R^{i}\left(\alpha \right)\in \mathrm{SO}\left(3\right)$ be the rotation matrix about the *i*-axis by the angle α with i∈{x,y,z} and α∈R.

Let $R_{AB}$ denote the transformation (rotation) matrix used to transform vectors from their representation in frame *B* to their representation in frame *A*, i.e.,

(A1) $${}_{A}\vec{r}=R_{AB}\cdot {}_{B}\vec{r}.$$

In this paper, whenever 2D kinematic vectors are shown, they are actually meant to be 3D vectors with the *y* component omitted to avoid overcrowding. The *y* component can be regarded as being 0 ∀t without loss of generality, as the motion of the pedal is constrained to the xz-plane.

### Appendix A.2. Pedal Kinematics

Given the world frame *I*, the pedal body frame *B*, the crank angle ϕ, and the pedal angle θ, as described in Section 3 and depicted in Figure 5, we can write the position of the pedal axle ${}_{I}\vec{x}$, represented in *I* as:

(A2) $${}_{I}\vec{x}=\left[\begin{array}{c} r\sin(\phi )\\ r\cos(\phi ) \end{array}\right]$$

where *r* is the distance between crank-arm axle and pedal axle, i.e., the crank arm length. Consequently, the velocity ${}_{I}\dot{\vec{x}}$ and the acceleration ${}_{I}\ddot{\vec{x}}$ can be written as:

(A3) $${}_{I}\dot{\vec{x}}=\left[\begin{array}{c} r\dot{\phi }\cos\left(\phi \right)\\ -r\dot{\phi }\sin\left(\phi \right) \end{array}\right]$$

(A4) $${}_{I}\ddot{\vec{x}}=\left[\begin{array}{c} -r{\dot{\phi }}^{2}\sin\left(\phi \right)+r\ddot{\phi }\cos\left(\phi \right)\\ -r{\dot{\phi }}^{2}\cos\left(\phi \right)-r\ddot{\phi }\sin\left(\phi \right) \end{array}\right]$$

by constraining ourselves to scenarios where $\ddot{\phi }\approx 0$, and by taking gravity into consideration, the acceleration ${}_{I}\ddot{\vec{x}}$ becomes:

(A5) $${}_{I}\ddot{\vec{x}}=\left[\begin{array}{c} -r{\dot{\phi }}^{2}\sin\left(\phi \right)\\ -r{\dot{\phi }}^{2}\cos\left(\phi \right)-g \end{array}\right].$$

Now, in order to represent the accelerations in the pedal frame *B*, we define the rotation matrix from *I* to *B* $R_{BI}$ as

(A6) $$R_{BI}=\left[\begin{array}{cc} \cos(\theta ) & \sin(\theta )\\ \sin(\theta ) & -\cos(\theta ) \end{array}\right]$$

hence, the acceleration of the pedal expressed in the body frame ${}_{B}\ddot{\vec{x}}$ is:

$${}_{B}\ddot{\vec{x}}=R_{BI}\cdot {}_{I}\ddot{\vec{x}}=\left[\begin{array}{c} -r{\dot{\phi }}^{2}\left(\sin(\phi )\cos(\theta )+\cos(\phi )\sin(\theta )\right)-g\sin\left(\theta \right)\\ -r{\dot{\phi }}^{2}\left(\sin(\phi )\sin(\theta )-\cos(\phi )\cos(\theta )\right)+g\cos\left(\theta \right) \end{array}\right].$$

The squared acceleration magnitude $\ddot{x}$ is:

(A7) $${\left|\right|}_{I}\ddot{\vec{x}}{\left|\right|}_{2}^{2}={\left|\right|}_{B}\ddot{\vec{x}}{\left|\right|}_{2}^{2}=r^{2}{\dot{\phi }}^{4}+2gr{\dot{\phi }}^{2}\cos\left(\phi \right)+g^{2}$$

hence, for every revolution of the crank which can be seen to be approximately performed at a constant rate $\dot{\phi }$, the acceleration magnitude has the form $\ddot{x}=\alpha \cos\left(\phi \right)+\beta$ with α,β>0 and is therefore maximal at ϕ=0rad and minimal at ϕ=πrad.

It shall be noted that the requirement of each revolution being carried out at a constant rate $\dot{\phi }\left(t\right)=\mathrm{const}\;\forall \;t\in \left[t_{{\mathrm{TDC}}_{i}},t_{{\mathrm{TDC}}_{i+1}}\right]$ is weaker than the requirement of steady state pedalling throughout a session $\dot{\phi }\left(t\right)=\mathrm{const}\;\forall \;t$.

Moreover, it shall be noticed that the pedal, i.e., the IMU in our system, experiences only translations (imagine keeping the pedal flat w.r.t. the ground and rotating the crank: the sensor’s axes keep aligned with the world frame, meaning that there is no rotation at all—the gyroscope will readout 0—despite the pedal moving on a circle) in addition to an oscillation about its axis (pedal angle θ), which is the source for the non-zero gyroscope measurements.

### Appendix A.3. A Note on the Chosen Frames of Reference and the Transformation Matrix R BI

The careful reader will have observed that the depicted frames *I* and *B* cannot be related by a rotation matrix $R_{IB}^{⊤}=R_{BI}\in \mathrm{SO}\left(2\right)$, as one would expect for two orthonormal frames. This can be dealt with by expanding the frames of reference to 3D, and by applying a first rotation of πrad about the *x*-axis when going from *I* to *B*. This will cause the two frames *I* and *B* to have their *y*-axes pointing in opposite directions. In fact, if we multiply the rotation matrix $R^{x}\left(\alpha \right)\in \mathrm{SO}\left(3\right)$ describing the first rotation about *x* by the angle α with α=πrad with the rotation matrix $R^{y}\left(\beta \right)\in \mathrm{SO}\left(3\right)$ describing the second rotation about *y* by β, we get:

(A8) $$\begin{array}{c} R:=R^{x}\left(\pi \right)R^{y}\left(\beta \right)=\\ =\left[\begin{array}{ccc} 1 & 0 & 0\\ 0 & -1 & 0\\ 0 & 0 & -1 \end{array}\right]\left[\begin{array}{ccc} \cos(\beta ) & 0 & \sin(\beta )\\ 0 & 1 & 0\\ -\sin(\beta ) & 0 & \cos(\beta ) \end{array}\right]=\left[\begin{array}{ccc} \cos(\beta ) & 0 & \sin(\beta )\\ 0 & -1 & 0\\ \sin(\beta ) & 0 & -\cos(\beta ) \end{array}\right] \end{array}$$

which one can see matches the definition of $R_{BI}$ given in (A6) with an added dimension (*y*). We could write all kinematics in 3D with the *y* components constrained to 0, but we opted for the more compact 2D notation. It shall be noted that this choice is debatable as it seems, as the right-handed system *I* is transformed into a left-handed system *B*, due to the fact that the 3D rotation corresponds to a 2D reflection, but one can always imagine the third component to be present but set to 0, and *I* and *B* have ${\vec{e}}_{y}^{B}=-{\vec{e}}_{y}^{I}$, which ‘restores’ *B* to a right-handed system.
